# Growing a Specialty-Specific Community of Practice in Education Scholarship

**DOI:** 10.5811/westjem.2015.9.28644

**Published:** 2015-10-22

**Authors:** Jeffrey N. Love, Douglas S. Ander

**Affiliations:** * Georgetown University School of Medicine, Department of Emergency Medicine, Washington, D.C.; † Emory University School of Medicine, Department of Emergency Medicine, Atlanta, Georgia

Emergency medicine (EM) educators have many masters. These include our hospital administrations who expect efficient patient care reflecting the priorities of safety and quality,^1^,^2^ the accreditation council for graduate medical education which has introduced a new competency-based standard by which our learners must be educated^3^,^4^ and last but not least, our learners that are using new educational modalities based on expanding digital platforms.^5^ To be successful, educators must satisfy each of these masters against the backdrop of increasing regulations, decreasing funding^6^,^7^ and information technology that appears to decrease our time with patients and perhaps learners in clinical practice.^8^

Success in our mission as educators is dependent upon coming together as a community of practice driven by scholarship that provides rigorous, high-impact studies that guide our educational practices. According to Lave and Wenger, a community of practice is defined as “groups of people who share a concern or a passion for something they do and learn how to do it better as they interact regularly.”^9^ Though there are a number of accomplished EM education researchers in the United States today, the specialty remains early in its development of such a community of practice. The Council of EM Residency Directors (CORD), Clerkship Directors of EM (CDEM) and the Society of Academic EM (SAEM) have all contributed to nascent progress in this regard.

For many years, SAEM/CDEM and CORD have offered presentations and abstracts related to education scholarship at the annual CORD Academic Assembly and SAEM meetings. Over the past seven years, the American Association of Medical Colleges and CORD have collaborated in a venture to provide a year-long experience in education research called the Medical Education Research Certification (MERC) at CORD Scholars’ Program.^10^,^11^ This program is based on the experiential learning theory of Kolb and the importance of social learning that is promoted through small group projects and multifaceted exercises. To date, 186 EM educators have participated in the program. A program evaluation of MERC at CORD over the first five years demonstrates that it has succeeded in (1) improving the education research skills and knowledge of participants, (2) resulted in subsequent research projects with peers from the program, (3) promoting the growth of a community of practice that supports its members both within the area of scholarship and beyond, and (4) contributed to participants earning new leadership positions in education.^12^

The CORD Academy of Scholarship is in the process of creating opportunities that build upon the MERC at CORD program. First and foremost, there is an ongoing effort to create a collaborative education research consortium to establish benchmarks/best practices while creating and disseminating new knowledge and related applications. In addition to serving as a central source of multicenter study administration, the consortium plans to encourage collaboration. Due to the multi-institutional design of these studies, the results are more likely to generalize to similar programs/institutions. Junior faculty will be encouraged to participate with more experienced members on projects. With increasing responsibilities and experience over time the intention is to facilitate the development of growing community of education scholars. Through these efforts both MERC at CORD and the CORD Academy continue to contribute to this developing community of practice by facilitating interested faculty’s ability to overcome individual obstacles to a career in scholarship including the lack of expertise, difficulty in identifying mentors, and a supportive network that promotes the professional growth of its members.^10^,^13^–^18^

Developing a successful career in education scholarship is also dependent upon a number of factors beyond the control of the individual. Institutional barriers to professional development are much more problematic including lack of protected time for scholarly pursuits, financial support and departmental recognition of related accomplishments. Such obstacles directly result from a lack of funding available for education scholarship. Working together, CORD and the EM Foundation have created a series of education research grants aimed at increasing the value of education research. Applications for these grants became available in October of 2015.

Most recently, CDEM and CORD have collaborated to develop and introduce this inaugural edition of the WestJEM Education Supplement. We hope to build this supplement into a regular forum that explores the breadth and depth of education scholarship as it relates to EM by sharing research findings, innovations, opinions of national experts and regular updates on state-of-the-art concepts in medical education. The ability to serve as a reviewer for this supplement provides an additional opportunity to hone interested faculty’s knowledge of the theory and principles that guide quality education scholarship. By providing a forum that values and cultivates education scholarship we hope to promote its growth and integration into the fabric of our specialty. The 107 manuscripts submitted for consideration are testimony to the enthusiasm and need for such a platform in our community. Ultimately, we strive to take another step in the direction of creating a community of practice centered on scholarship that will allow EM educators to grow and prosper.
